# Drug Susceptibility Patterns in MDR-TB Patients: Challenges for Future Regimen Design. A Cross-Sectional Study

**DOI:** 10.1371/journal.pone.0142425

**Published:** 2015-11-11

**Authors:** Helen R. Stagg, James Brown, Elmira Ibraim, Vija Riekstiņa, Piret Viiklepp, Andra Cīrule, Horia Cocei, Manfred Danilovitš, Gunta Dravniece, Charlotte Jackson, Peter J. White

**Affiliations:** 1 Research Department of Infection and Population Health, University College London, London, United Kingdom; 2 Centre for Respiratory Medicine, Royal Free London NHS Foundation Trust, London, United Kingdom; 3 Division of Medicine, University College London, London, United Kingdom; 4 Marius Nasta Institute of Pulmonology, Bucharest, Romania; 5 Centre of TB and Lung Diseases, Riga East University Hospital, Riga, Latvia; 6 University of Latvia, Riga, Latvia; 7 Estonian Tuberculosis Registry, National Institute for Health Development, Tallinn, Estonia; 8 Lung Clinic, Tartu University Hospital, Tartu, Estonia; 9 KNCV Tuberculosis Foundation, The Hague, The Netherlands; 10 MRC Centre for Outbreak Analysis & Modelling and NIHR Health Protection Research Unit in Modelling Methodology, Department of Infectious Disease Epidemiology, Imperial College School of Public Health, London, United Kingdom; 11 Modelling and Economics Unit, Centre for Infectious Disease Surveillance and Control, Public Health England, London, United Kingdom; University of Padova, Medical School, ITALY

## Abstract

Globally, there is substantial concern regarding the challenges of treating complex drug resistance patterns in multidrug resistant tuberculosis cases. Utilising data from three different settings (Estonia, Latvia, Romania) we sought to contrast drug susceptibility profiles for multidrug resistant tuberculosis cases, highlight the difficulties in designing universal regimen, and inform future regimen selection. Demographic and microbiological surveillance data for multidrug resistant tuberculosis cases from 2004–13 were analysed. High levels of additional resistance to currently recommended second line drugs were seen in all settings, with extensive variability between countries. Accurate drug susceptibility testing and drug susceptibility testing data are vital to inform the development of comprehensive, flexible, multidrug resistant tuberculosis guidance.

## Introduction

The World Health Organization (WHO) estimates that 480,000 people globally had incident multidrug resistant tuberculosis (MDR-TB) in 2013; 97,000 started MDR-TB treatment [1]. The global treatment success rate for the 2011 cohort of patients was 48% [1]. 20 month regimens with an intensive phase of 8 months are recommended for most patients that contain at least four second-line drugs likely to be effective in the intensive phase (“a fluoroquinolone, a parenteral agent, ethionamide (or prothionamide), and either cycloserine or *p*-aminosalicylic acid (PAS)”), as well as the first-line drug pyrazinamide [2]. Tailoring treatment regimens based on individual resistance patterns, which may be highly complex in MDR-TB patients, improves the chance of cure, but may not always be feasible.

Estonia, Latvia and Romania are three central European countries within the WHO European region with contrasting challenges for MDR-TB control. Estonia and Latvia are both ranked as high MDR-TB burden countries, with an estimated 17% of new tuberculosis (TB) cases and 48% of retreatment cases MDR-TB in Estonia, and 9% and 26%, respectively, in Latvia, in 2013 [1]. Estonia is classified as having a high HIV burden, with 13% of tested TB patients HIV positive [1]. In Romania the percentage MDR-TB was estimated to only be 3% among new tuberculosis (TB) cases and 11% among retreatment cases in 2013, but Romania’s high TB incidence and population size mean absolute numbers of pulmonary MDR-TB cases in 2013 were 4.5 times greater than in Estonia and Latvia combined [1].

By describing and contrasting drug resistance patterns among MDR-TB patients in three nations with differing epidemiological profiles and for whom MDR-TB is of concern, as well as reflecting on these patterns in the light of WHO MDR-TB treatment guidance, we sought to inform the development of future treatment recommendations applicable to a variety of settings, without being adversely prescriptive.

## Methods

For this cross-sectional study demographic and microbiological data for diagnosed and notified MDR-TB cases were extracted from the three countries’ surveillance, clinical and laboratory systems for the years 2004–2013. An effort was made to access all cases and case data, in order to avoid bias. This time frame represented the most recent decade for which data were available from all three countries.

Drug susceptibility testing (DST) results for the first line drugs ethambutol, isoniazid, pyrazinamide and rifampicin; the injectables amikacin, capreomycin and kanamycin; the fluoroquinolones ofloxacin and moxifloxacin; the oral second line bacteriostatic drugs cycloserine, p-aminosalicylic acid (PAS) and prothionamide; and the second/third line drug linezolid were available for 2013. Streptomycin is not recommended for use in MDR-TB cases by WHO and hence was not included [2].

During the time period studied Estonia used BACTEC MGIT as its gold standard DST for first line drugs and capreomycin, amikacin, kanamycin, prothionamide, ofloxacin, moxifloxacin, and linezolid. (HAIN GenoType MTBDRplus is also used as a preliminary diagnostic for isoniazid and rifampicin and MTBDRsl for fluoroquinolones and capreomycin, amikacin and kanamycin, but if there is a discrepancy with the BACTEC MGIT results the latter are taken.) Latvia used the indirect proportion method on Löwenstein-Jensen solid media for isoniazid, rifampicin, ethambutol, kanamycin, amikacin, capreomycin, ofloxacin, ethionamide (as representative of both ethionamide and prothionamide sensitivity testing), cycloserine, and PAS and indirect DST in the BACTEC MGIT system for isoniazid, rifampicin, pyrazinamide, amikacin, capreomycin, and ofloxacin. The choice of method for isoniazid and rifampicin depended upon whether the patient was deemed high risk for MDR-TB; if this was the case BACTEC MGIT is chosen. In (rare) instances of disagreement for ofloxacin sensitivity the worst case scenario, i.e. resistance, was assumed. GeneXpert was introduced in Estonia in 2009 and Latvia in 2010; rifampicin sensitivity results are always confirmed by culture in both countries. Romania used Löwenstein-Jensen solid media for isoniazid, rifampicin, ethambutol, kanamycin, amikacin, capreomycin, ofloxacin, ethionamide (as representative of both ethionamide and prothionamide sensitivity testing), cycloserine, and PAS. Genetic tests and DST on liquid media have been used in few laboratories for selected cases (with a high probability of MDR-TB). In case of discordant results, a decision was taken based on DST on the solid media results.

New (a ‘patient who has never been treated for TB or has taken anti-TB drugs for less than one month’) and retreatment (a ‘patient who has been treated for one month or more with anti-TB drugs in the past’) cases, defined as per WHO guidance [1], were analysed separately. The percentage of MDR-TB cases known to be fluoroquinolone resistant, fluoroquinolone and/or second-line injectable resistant, and extensively drug resistant (XDR; both fluoroquinolone and second-line injectable resistant) was examined over time. For these calculations ofloxacin was the only fluoroquinolone considered in order to ensure consistency between the countries, as Latvia and Romania did not test moxifloxacin sensitivity.

Data were additionally broken down by population group- children under 14 years of age, individuals with HIV, and vulnerable individuals (unemployed, drug users, alcoholics, and/or previous or current imprisonment).

This study did not undertake primary research on human subjects; it utilised aggregate, pre-collected, surveillance data that was anonymised and de-identified in-country prior to analysis and thus did not require informed consent. All countries have ethical permission to collect surveillance data and analyse it under such conditions without further study-specific ethical approvals being required (Estonia: Government Regulation Act for Tuberculosis Registry No 70, 26.05.2011; Romania: Ministry of Health for Romania 'Technical norms for national public health programs implementation'; Latvia: approval for this project from the ethics committee of Riga Stradins University).

## Results

In 2013 Estonia had 50 MDR-TB cases (29 new and 21 retreatment), Latvia 76 (50, 26) and Romania 575 (165, 410) (Tables 1 and 2). In Estonia and Latvia the vast majority of cases underwent second line DST, but only approximately two thirds in Romania. In both Estonia and Latvia a large percentage (~50%) of MDR-TB cases were fluoroquinolone and/or second-line injectable resistant; this percentage was lower in 2013 than 2004. A similar percentage of MDR-TB cases were XDR in Latvia and Estonia in 2013, but only in Latvia does this percentage appear to have been increasing since 2004 (Table 1). The large increase in Romania (6.8-fold change in percentage) represents changes in sensitivity testing and reporting for fluoroquinolones and second line injectables in 2007. Accounting for such changes by using 2007 as the baseline for Romania, the percentage of MDR-TB cases that are fluoroquinolone and/or second-line injectable resistant may be stable (1.1-fold change between 2007 and 2013). In Romania more MDR-TB cases are resistant to second-line injectables than fluoroquinolones (57% in 2013); the reverse is true in Estonia (24% in 2013). For all countries in 2013 versus 2004 resistance to injectables proportionally accounted for less of the additional resistance among MDR-TB cases than resistance to fluoroquinolones.

**Table 1:** Additional drug resistance in multidrug resistant tuberculosis patients in Estonia, Latvia and Romania in 2013.

	Estonia	Latvia	Romania
	# (% of MDR-TB, fold change in % since 2004)	# (% of MDR-TB, fold change in % since 2004)	# (% of MDR-TB, fold change in % since 2004)
Total MDR-TB cases	50	76	575
Resistant to fluoroquinolones and/or injectables	25 (50.0%, 0.7)	36 (47.4%, 0.8)	143 (24.9%, 6.8^a^)
Resistant to fluoroquinolones	19 (38.0%, 1.4)	17 (22.4%, 1.7)	61 (10.6%, 27.9^a^)
XDR	8 (16.0%, 0.8)	13 (17.1%, 1.7)	38 (6.6%, 52.1^a^)

^a^DST testing and reporting changed in Romania in 2007, resulting in these large fold changes in percentages. By comparison, the respective fold changes since 2007 are: resistant to fluoroquinolones and/or injectables 1.1, resistant to fluoroquinolones 1.3, XDR 1.0. MDR-TB- multi-drug resistant tuberculosis; resistant to fluoroquinolones- ofloxacin only for consistency between countries; XDR- extensively drug resistant.

**Table 2:** Drug susceptibility testing results for multidrug resistant tuberculosis patients in Estonia, Latvia and Romania in 2013.

	Estonia		Latvia		Romania	
	New cases	Retreatment cases	New cases	Retreatment cases	New cases	Retreatment cases
	# (%)	# (%)	# (%)	# (%)	# (%)	# (%)
Total	29	21	50	26	165	410
Number undergoing 1^st^ line DST	29 (100.0)	21 (100.0)	50 (100.0)	26 (100.0)	165 (100.0)	410 (100.0)
Additional resistance to…						
Ethambutol	27 (96.4)^a^	20 (95.2)	36 (72.0)	21 (80.8)	68 (70.1)^b^	195 (76.5)^b^
Pyrazinamide	20 (71.4)^a^	15 (71.4)	36 (75.0)^c^	18 (69.2)	Not tested	Not tested
Number undergoing 2^nd^ line DST	28 (96.6)	21 (100.0)	50 (100.0)	26 (100.0)	108 (65.5)	276 (67.3)
Additional resistance to…						
Amikacin	3 (10.7)	3 (14.3)	22 (44.0)	9 (34.6)	9 (13.6)^b^	51 (33.1)^b^
Capreomycin	3 (10.7)	3 (14.3)	25 (50.0)	15 (57.7)	13 (18.6)^b^	61 (37.2)^b^
Kanamycin	5 (17.9)	9 (42.9)	21 (42.9)^d^	15 (57.7)	18 (20.7)^b^	94 (40.2)^b^
Ofloxacin	10 (35.7)	9 (42.9)	9 (18.0)	8 (30.8)	13 (18.8)^b^	48 (31.6)^b^
Moxifloxacin	2 (10.5)^e^	2 (16.7)^e^	Not tested	Not tested	Not tested	Not tested
Cycloserine	Not tested	Not tested	1 (2.0)	0 (0.0)	2 (7.1)^b^	17 (16.2)^b^
PAS	Not tested	Not tested	12 (25.0)^c^	5 (19.2)	5 (8.3)^b^	14 (9.3)^b^
Prothionamide	4 (14.3)	9 (42.9)	26 (52.0)^f^	19 (73.1)^f^	10 (11.2)^f^	27 (12.3)^f^
Linezolid	0 (0.0)^g^	2 (14.3)^g^	Not tested	Not tested	Not tested	Not tested

^a^Denominator 28

^b^denominators (new cases, retreatment cases) ethambutol 97, 255; amikacin 66, 154; capreomycin 70, 164; kanamycin 87, 234; ofloxacin 69, 152; cycloserine 28, 105; PAS 60, 151

^c^denominator 48

^d^denominator 49

^e^denominator 19 new, 12 retreatment

^f^ethionamide DST undertaken in Latvia and Romania (denominators for new Romanian cases 89, retreatment cases 220)

^g^denominator 21 new, 14 retreatment. DST- drug susceptibility testing; PAS- p-aminosalicylic acid.

Drug resistance patterns for cases diagnosed in 2013 were broken down further, as would be required to inform treatment decisions (Table 2). Resistance to the first-line drugs pyrazinamide and ethambutol was high (70–96% resistant to ethambutol across all cases and countries; 69–75% resistant to pyrazinamide in Estonia and Latvia (not tested for in Romania)).

Only Estonia undertook DST to moxifloxacin as well as ofloxacin among the fluoroquinolones (Table 2); of 19 ofloxacin resistant cases 16 were tested for susceptibility to moxifloxacin and four found to be resistant. The percentage of MDR-TB cases resistant to ofloxacin was high in Estonia and for retreatment cases in Latvia and Romania.

Resistance patterns to the injectables capreomycin and amikacin were relatively uniform between new and retreatment cases, apart from in Romania, with Latvian cases and Romanian retreatment cases most likely to be resistant (Table 2). In all three countries kanamycin resistance was more common in retreatment cases than in new cases. In Estonia kanamycin resistance was more common than resistance to other injectables; of the 14 cases resistant to kanamycin six were also resistance to amikacin and capreomycin.

Romania and Latvia also undertook DST to cycloserine and PAS (Table 2). PAS and cycloserine resistance are of particular concern for Romanian retreatment cases, and PAS resistance for all Latvian cases. Prothionamide/ethionamide resistance was high among Latvian cases and Estonian retreatment cases, but not elsewhere.

Only Estonia undertook DST to the group 5 drug linezolid [3]; resistant cases were few in number (Table 2).

Latvia sees the highest percentage of MDR-TB cases (24% in 2013) co-infected with HIV, with 96%, 91% and 75% of MDR-TB cases tested in Estonia, Latvia and Romania, respectively. Paediatric MDR-TB was rare in all countries. Vulnerable population groups comprised 74% of MDR-TB cases in Estonia, and 54% in Romania, in 2013 (data unavailable for Latvia).

## Discussion

Extensive additional drug resistance exists in MDR-TB cases from Estonia, Latvia and Romania, with considerable between-country variability. It is useful to evaluate such patterns in relation to WHO treatment recommendations for MDR-TB: the high percentage of cases pyrazinamide resistant means that this drug is unlikely to benefit many patients; the increase in the proportion of cases with additional resistance to fluoroquinolones; high levels of resistance to prothionamide/ethionamide in Latvia cases and Estonian retreatment cases. The difference in the resistance profiles of MDR-TB cases in Estonia and Latvia is especially interesting, given their geographical proximity.

Our greatest uncertainty surrounds the estimates for Romania, given the low percentage of cases with a reported 2^nd^ line DST and concerns about their representativeness. It should also be borne in mind that notified cases in Romania excluded those that were not treated e.g. due to broad drug resistance. Reporting fold changes may conceal more complex trends; however, except where noted, these figures were consistently decreasing over the study period.

It is critical in the context of any report that utilises phenotypic or genotypic DST data to consider the reliability and reproducibility of the tests utilised.[4] On the basis of available evidence the WHO classifies DST for moxifloxacin, cycloserine, PAS, and prothionamide as category four (of a possible five, with routine DST not being recommended for category four or five); linezolid DST to category five and ofloxacin DST to category three. The BACTEC MGIT testing system may overestimate the likelihood of pyrazinamide resistance.[5] All three countries used phenotypic DST, which provides reassurance about the comparability of the data, however the assessed levels of additional drug resistance in the described MDR-TB patients may be inaccurate for certain drugs due to the quality of the tests available. Thus the usefulness of such testing data for clinical decision making should be carefully evaluated.

Dalton et al. and Kurbatova et al. have reported data from the Preserving Effective TB Treatment Study (PETTS), a cohort of adults (including individuals from Estonia and Latvia) with pulmonary MDR-TB who started treatment with second-line drugs 2005–2008 [6,7]. Although both papers examined additional drug resistance in MDR-TB cases (although not reporting separately for new and retreatment cases), Kurbatova pooled data across continents. The most recent entry to the cohort was five years previous to our dataset and our population-based dataset may be more representative than PETTS participants, which may explain the striking contrasts in terms of a much lower proportion of cases resistant to capreomycin in Latvia, and a suggestion of lower levels of ethambutol and fluoroquinolone resistance in the latter [6]. Zignol et al. previously analysed surveillance data of drug resistance patterns across Europe 1997–2012, broadly examining resistance to fluoroquinolones and/or injectables for Estonia and Latvia, but not in the detail provided here [8].

The implementation of an individualised regimen policy within a country requires high quality DST data and a fast turnaround time. Given the relatively small number of cases in Estonia and Latvia it may be feasible to individualise treatment regimens. In Romania individualisation was more challenging as capreomycin and PAS were only available through the Global Fund for certain patients. Between-country differences complicate future guideline development for treatment regimens and drug susceptibility surveillance. The complexity of the resistance patterns reported makes treating cases using programmatic guidelines in the absence of 2^nd^ line DST results very difficult and risks cases accumulating further resistance if access to second line or new drugs increases without improved DST access.

By describing and comparing the patterns of additional drug resistance of MDR-TB cases in three epidemiologically distinct nations we illuminate the difficulties surrounding the development of future treatment guidelines, and highlight some particular areas of concern for MDR-TB control in Estonia, Latvia and Romania for the coming years. Accurate DST and DST data for second line drugs from a variety of countries is vital for comparison to WHO treatment recommendations in order to inform the development of future MDR-TB treatment regimens that meet the needs of all regions and patient groups.

## Disclaimer

The views expressed are those of the authors and not necessarily those of the UK Medical Research Council, National Health Service, National Institute for Health Research, Department of Health, or Public Health England.
